# Transcriptome remodeling drives acclimation to iron availability in the marine N_2_-fixing cyanobacterium *Trichodesmium erythraeum* IMS101

**DOI:** 10.1128/msystems.01499-24

**Published:** 2025-04-17

**Authors:** Xin Zhong, Ran Duan, Shengwei Hou, Meng Chen, Xiaoming Tan, Wolfgang R. Hess, Tuo Shi

**Affiliations:** 1 Marine Genomics and Biotechnology Program, Institute of Marine Science and Technology, Shandong University, Qingdao, Shandong, China; 2 State Key Laboratory of Marine Environmental Science, College of Ocean and Earth Sciences, Xiamen University 534813 , Xiamen, Fujian, China; 3 Department of Ocean Science and Engineering, Southern University of Science and Technology 547494 , Shenzhen, Guangdong, China; 4 Genetics and Experimental Bioinformatics, Institute of Biology III, University Freiburg, Freiburg, Germany; 5 State Key Laboratory of Biocatalysis and Enzyme Engineering, Environmental Microbial Technology Center of Hubei Province, School of Life Sciences, Hubei University 117923 , Wuhan, Hubei, China; University of Illinois Chicago, Chicago, Illinois, USA

**Keywords:** *Trichodesmium*, iron limitation, transcriptome remodeling, *isiA *gene cluster, marine diazotroph

## Abstract

While enhanced phytoplankton growth as a result of iron (Fe) fertilization has been extensively characterized, our understanding of the underlying mechanisms remains incomplete. Here, we show in a laboratory setup mimicking Fe fertilization in the field that transcriptome remodeling is a primary driver of acclimation to Fe availability in the marine diazotrophic cyanobacterium *Trichodesmium erythraeum* IMS101. Fe supplementation promoted cell growth, photosynthesis and N_2_ fixation, and concomitant expression of the photosynthesis and N_2_ fixation genes. The expression of genes encoding major Fe-binding metalloproteins is tightly linked to cellular carbon and nitrogen metabolism and appears to be controlled by the ferric uptake regulator FurA, which is involved in regulating Fe uptake and homeostasis. This feedback loop is reinforced by substitutive expression of functionally equivalent or competitive genes depending on Fe availability, as well as co-expression of multiple Fe stress inducible *isiA* genes, an adaptive strategy evolved to elicit the Fe-responsive cascade. The study provides a genome-wide perspective on the acclimation of a prominent marine diazotroph to Fe availability, reveals an upgraded portfolio of indicator genes that can be used to better assess Fe status in the environment, and predicts scenarios of how marine diazotrophs may be affected in the future ocean.

**IMPORTANCE:**

The scarcity of trace metal iron (Fe) in global oceans has a great impact on phytoplankton growth. While enhanced primary productivity as a result of Fe fertilization has been extensively characterized, the underlying molecular mechanisms remain poorly understood. By subjecting the model marine diazotroph *Trichodesmium erythraeum* IMS101 to increasing concentrations of supplemented Fe, we demonstrate in it a comprehensively remodeled transcriptome that drives the mobilization of cellular Fe for coordinated carbon and nitrogen metabolism and reallocation of energy and resources. Our data provide broad genomic insight into marine diazotrophs acclimation to Fe availability, enabling the versatility and flexibility in choice of indicator genes for monitoring Fe status in the environment and having implications on how marine diazotrophs persist into the future ocean.

## INTRODUCTION

Cyanobacteria in the genus *Trichodesmium* are a prominent keystone N_2_-fixing taxon (i.e., diazotroph) that dominates in tropical and subtropical oceans and play a crucial role in global carbon and nitrogen cycles (1–4). The abundance and distribution of *Trichodesmium* are affected by many environmental factors including temperature (5–7), pH (8–10), and nutrients (11, 12), particularly the content of trace metal iron (Fe) (13–15). In the vast majority of the world’s open ocean, the concentration of dissolved Fe that can be directly used is extremely low due to limited access to airborne dust and the oxidized state of seawater (16, 17). The concentration of dissolved inorganic Fe (dFe') in surface water is approximately only one-millionth of the concentration of plankton cells (i.e., one million cells competing for one Fe atom available) (18), which severely limits the primary productivity of phytoplankton. The impact of Fe limitation on phytoplankton growth has been confirmed by a dozen “Fe fertilization” mesocosm experiments deliberately performed in the high nutrient and low chlorophyll regions (19–23), as well as phytoplankton growth fueled by haphazard, natural events such as volcanic eruptions (24–26). Controlled laboratory experiments also demonstrated that Fe limitation severely inhibited the physiological activities of diatoms (27, 28), dinoflagellates (29, 30), coccolithophores (31, 32), and diazotrophic cyanobacteria including *Trichodesmium* and *Crocosphaera* (33–36).

Fe is an indispensable redox component of several essential cellular processes, including photosynthesis and N_2_ fixation (37). In *Trichodesmium*, the simultaneous occurrence of these two processes during the light period (38) is puzzling, given their competition for intracellular Fe at the same time and the inhibition of nitrogenase by O_2_ produced during photosynthesis. One plausible explanation is the spatial segregation between morphologically similar vegetative and diazotrophic cells within the filaments of the non-heterocystous *Trichodesmium* (38, 39) though true “diazocytes” have not been definitively identified to date (40, 41). Other mechanisms to protect nitrogenase may be involved, for example, photosynthetic proteins in nitrogenase-containing cells being inactive, RuBisCO acting as an oxygenase (photorespiration) at noon, and cytochrome oxidase driving rapid O_2_ consumption (dark respiration) (40). Additionally, the potential presence of O_2_ diffusion barriers (38), accumulation of antioxidants (42), ROS elimination through the Mehler reaction, and variability in the composition of phycobiliproteins (43) also play important roles in regulating photosynthesis during N_2_ fixation. A recent study in natural populations of *Trichodesmium* has also shown that energy from photosynthesis can be shuttled directly to nitrogenase rather than to the glycogen production, thereby reducing the cells’ density and sinking rate while enhancing their ability to acquire Fe from atmospheric dust particles (44).

Studies have demonstrated that *Trichodesmium* has evolved specific strategies to acquire and eﬃciently use Fe under Fe-limited conditions (45–48), such as changing colony morphologies to effectively collect Fe-rich dust particles (49, 50), sacrificing N_2_-fixation to sustain photosynthesis (14), adjusting cell size and surface area (51), or reorganizing thylakoid membrane electron transport and changing cellular energy production pathways (52) to redistribute intracellular Fe resources. Proteomic analysis has revealed that changes in Fe availability reshape the Fe demand of *Trichodesmium*, resulting in approximately 50% less metabolic Fe needed under Fe-limited conditions (47), with some Fe-binding proteins being substituted by Fe-free equivalents (53). Transcriptomic analysis of both laboratory and field experiments has identified genes of Fe-stress biomarkers in N_2_ fixation, photosynthesis, and Fe transport and storage, the expression of which is progressively changed with decreasing Fe availability (46, 54). In addition, research into the influence of iron, phosphorus, and ocean acidification on *Trichodesmium* and the unicellular marine N_2_-fixing cyanobacterium *Crocosphaera* also shows that the diazotrophs could respond to the challenges posed by climate change and nutrient limitation at both transcriptional and translational levels (36, 53, 55, 56).

Despite the advances in previous studies, there remain many unanswered questions regarding *Trichodesmium* acclimation to Fe availability. Due to limited annotations of the *Trichodesmium* genome and a high number of genes of unknown functions, only a small number of biomarkers have so far been identified and characterized (54–56). Transcriptome-wide profiling of *Trichodesmium* response to variations in Fe availability may help open new avenues for searching genes and functions previously misannotated or overlooked. Moreover, the dynamic range of Fe concentrations in the future ocean is difficult to predict. For example, the drought and desertification driven by climate change, suboptimal agricultural activities, and the still increasing fossil fuel emissions are all expected to significantly increase inputs of continental dust to much of the future ocean (57, 58), whereas the increasingly acidified seawater could affect the uptake of Fe by marine microbes by altering the chelation degree of Fe with organic ligands (59). These circumstances suggest that marine diazotrophs such as *Trichodesmium* may have plastic regulatory mechanisms in response to the labile Fe concentrations in the environment, a topic that has not been fully explored. To address these issues, we cultivated the model marine diazotroph *Trichodesmium erythraeum* strain IMS101 (hereafter IMS101) under laboratory conditions mimicking open ocean Fe fertilization. By monitoring the physiological performance of IMS101 alongside transcriptomic analysis with the Illumina RNA-Seq platform, we found stimulated growth, photosynthesis and N_2_ fixation in IMS101 with increased Fe availability, and a comprehensively remodeled transcriptome associated with the mobilization of cellular Fe for coordinated carbon and nitrogen metabolism and reallocation of energy and resources. The study provides not only a genome-wide perspective toward acclimation to Fe availability in *Trichodesmium* and other marine diazotrophs but also an up-to-date portfolio of Fe-responsive indicator genes that will facilitate the monitoring of Fe availability status in the changing marine ecosystem.

## RESULTS

**Fig 1:**
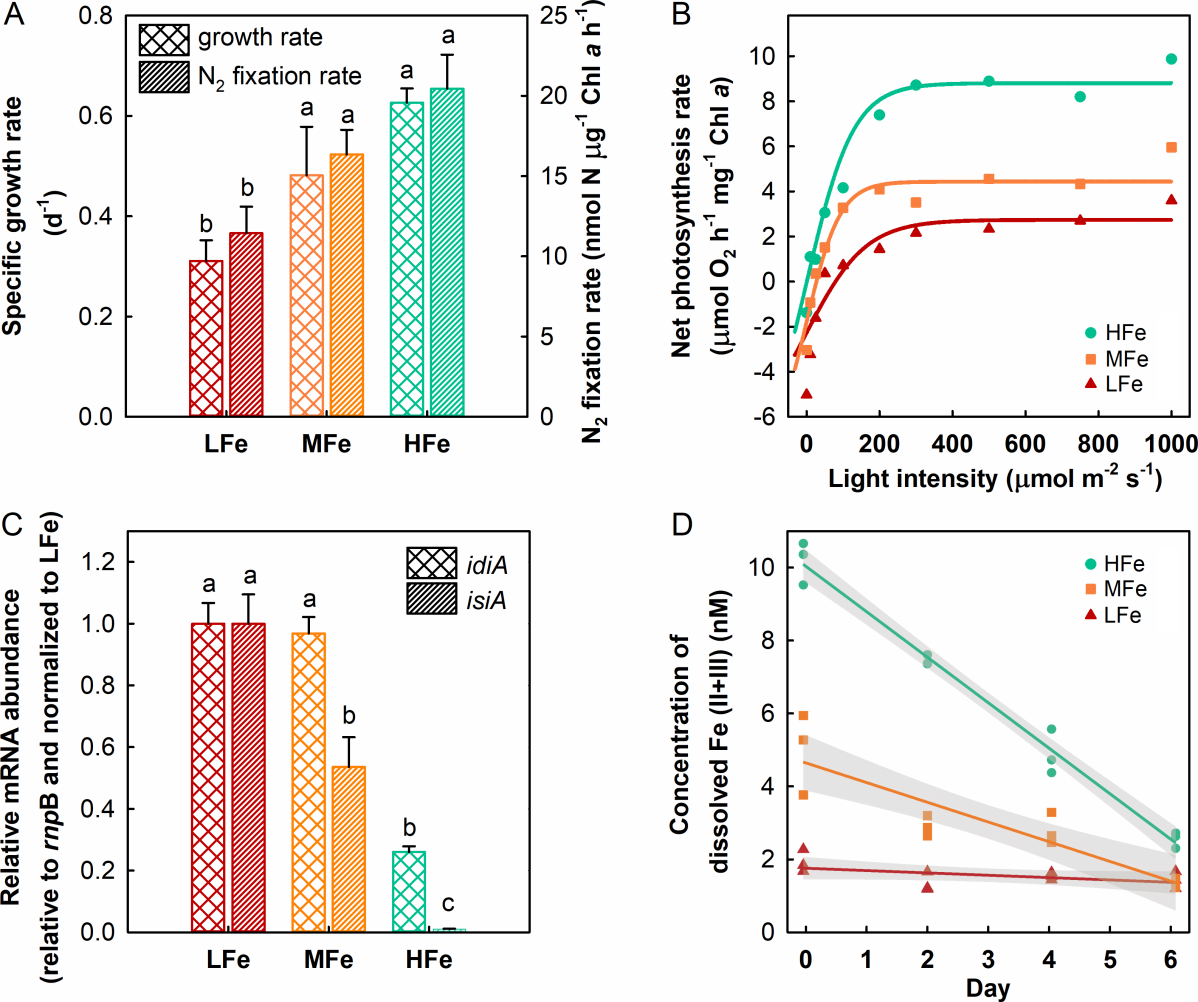
Physiological response of *T. erythraeum* IMS101 to increased iron availability. Shown are the chlorophyll-based specific growth rates and N_2_ fixation rates (**A**), photosynthesis-irradiation curve fitted with the rates of oxygen evolution (**B**), relative mRNA abundances of two iron (Fe) stress indicator genes, *idiA* and *isiA* (**C**), and measured concentrations of dissolved Fe redox species (II + III) (dFe') in the culture media at designated time points during IMS101 growth (**D**). Measurements of N_2_ fixation rates, photosynthetic O_2_ evolution, and qPCR assay of Fe stress genes were conducted using samples collected on days 6, 7, and 5, respectively. IMS101 was grown in media supplemented with 0, 10, and 100 nM of FeCl_3_ representing the low (LFe), medium (MFe), and high (HFe) concentration iron treatments, respectively. The data are plotted as mean ± standard deviation of biological triplicates (*n* = 3). Dissimilar lowercase letters in panels A and C represent significant differences across the treatments (*P* < 0.05). The shaded area in panel D represents the 95% confidence interval.

We set up a laboratory-controlled experiment to mimic the scenario of Fe fertilization in the field to investigate IMS101 acclimation to Fe availability. Three concentrations of FeCl_3_ at 0, 10, and 100 nM complexed with 20 µM ethylenediaminetetraacetic acid (EDTA) were applied to represent the low (LFe), medium (MFe), and high (HFe) concentration Fe supplemented, respectively. The measured initial concentrations of dissolved Fe (dFe') were approximately 2, 5, and 10 nM for the stock media LFe, MFe, and HFe, respectively, and the concentrations of dFe' decreased with time during IMS101 growth (Fig. 1). While stringent trace metal clean techniques were not applied, we were able to properly manipulate Fe limitation and fertilization in the laboratory setting here, as seen from the significant differences in specific growth rate, consumption of dFe', and expression of the Fe stress genes across the Fe supplementation treatments (Fig. 1; Fig. S1). It is also worth noting that the dFe' concentration in the LFe medium was higher than the level in nature that is often critically limiting (60). However, given the much higher abundance of IMS101, the LFe culture can arguably be considered Fe-limited, explaining the observations in this study.

### Physiological responses of IMS101 to Fe supplementation

Compared with the LFe (i.e., Fe-deplete) control, the chlorophyll *a* (Chl *a*)-based specific growth rates (μ) of IMS101 were 1.5 and 2 times increased under the MFe and HFe treatments, respectively. Similarly, the N_2_ fixation rates were increased 1.5 to 2 times under Fe-replete conditions (MFe and HFe) (Fig. 1A). The maximum light utilization coefficient (*α*) and maximum photosynthetic rate (*P*_max_) were 2.19–2.22 and 1.2–1.9 times increased under the MFe and HFe conditions, respectively (Fig. 1B). In addition, the *idiA (Tery_3377*) transcript levels were increased 3.7 and 3.9 times under the MFe and LFe treatments, respectively, relative to that under the HFe condition (Fig. 1C). The transcript levels of the *isiA* (*Tery_1667*) gene were increased 51 and 96 times under the MFe and LFe treatments, respectively (Fig. 1C). The consumption of Fe was the fastest in the HFe treatment and the slowest in the LFe treatment (Fig. 1D), suggesting that IMS101 can utilize Fe and the Fe consumption is positively correlated with growth rate.

### Overall transcriptomic remodeling of IMS101

**Fig 2:**
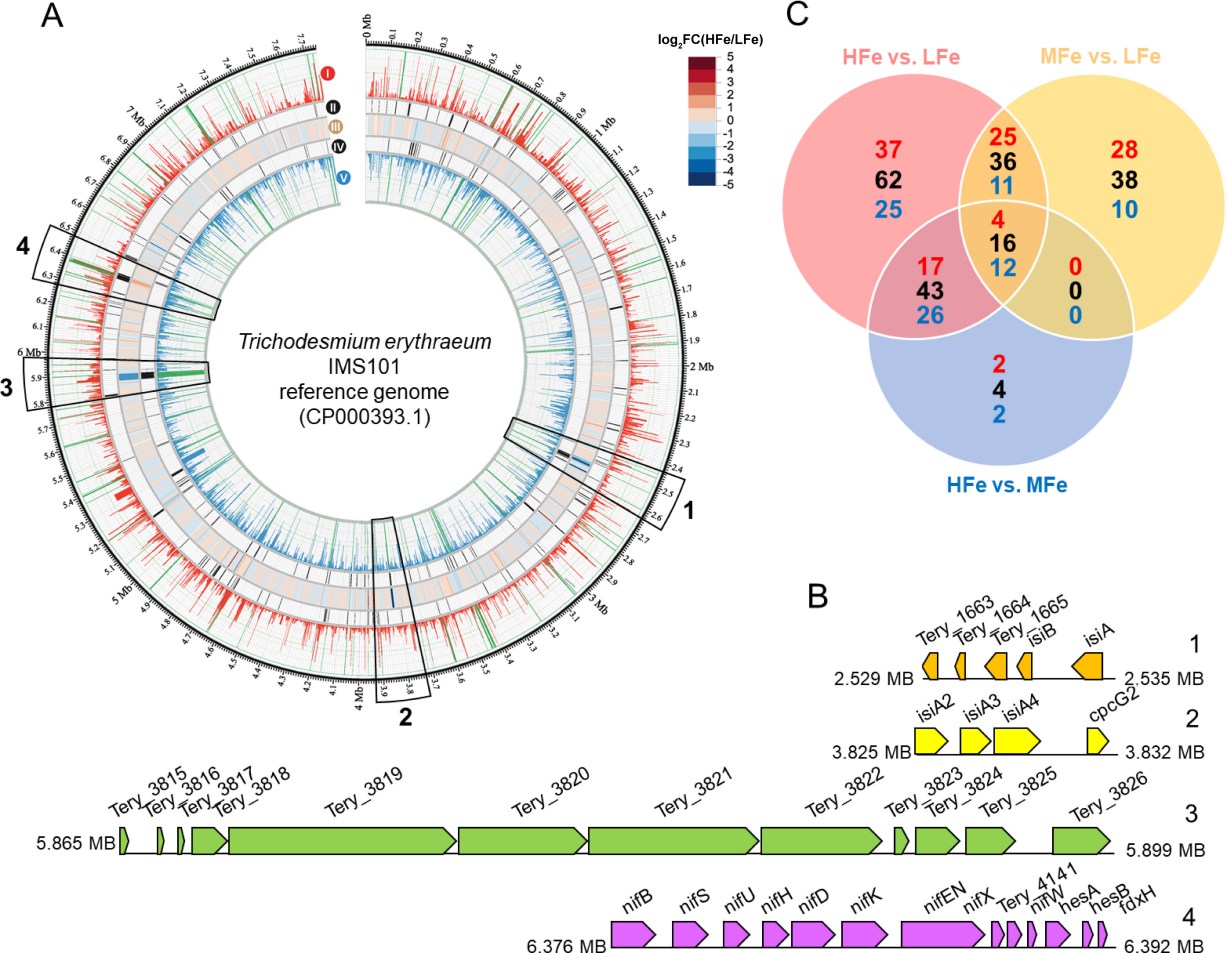
Transcriptome landscape of *T. erythraeum* IMS101 in acclimation to increased iron availability. (**A**) Circular presentation of differentially expressed genes between the HFe and LFe conditions along the IMS101 genome. The rings of the circos plot from the outermost to the innermost are (i) average read count per gene of the HFe treated samples, (ii) significantly upregulated genes under the HFe condition highlighted in dark color, (iii) heatmap of log_2_FC between the HFe and LFe conditions, (iv) significantly downregulated genes under the HFe condition highlighted in dark color, and (v) average read count per gene of the LFe treated samples. For visualization purposes, a maximum read count of 50,000 has been set for rings i and v, and counts above this threshold are highlighted with green bars. Log_2_FC, log_2_-scaled fold change between the HFe and LFe treatments after normalization. (**B**) Close-up view of the four gene clusters depicted in panel A, the downregulated *isiAB* operon (1), *isiA2-isiA3-isiA4-cpcG2* (2), a cryptic gene cluster possibly involved in anti-microbial secondary metabolite biosynthesis (3), and the upregulated nitrogenase *nif* gene cluster (4). (**C**) Venn diagram summarizing the numbers of differentially expressed genes among treatments. Numbers in black represent the number of genes with >2-fold changes. From these, the numbers of upregulated genes are shown in red and those of downregulated genes in blue.

Using the high-throughput Illumina RNA-Seq sequencing technique, we performed genome-wide gene expression analysis in IMS101 corresponding to Fe supplementation (Table S1). Of the 4,989 protein-coding genes in the IMS101 reference genome (61) (https://www.ncbi.nlm.nih.gov/nuccore/CP000393.1/), a total of 199 genes were differentially expressed following the criterion of log_2_-transformed fold change (log_2_FC) >1 and adjusted *P*-value (padj) < 0.05 in the pairwise comparison between different treatments (Fig. S2; Table S2). The numbers of differentially expression genes (DEGs) under the HFe vs LFe, MFe vs LFe, and HFe vs MFe comparisons were 157, 90, and 63, respectively, and the numbers of up and downregulated genes across all comparisons were 113 and 86, respectively (Fig. 2; Fig. S3). In the HFe/LFe treatment comparison, the top 10 most significantly upregulated genes were the genes encoding small heat shock protein Hsp20 (*hsp20*, *Tery_3061/3062/3063/3064*), Fe-S binding ferredoxin (*fdxB*), cytochrome *c_6_* (*petJ*), B_12_-independent methionine synthase (*metE*), H_2_ uptake hydrogenases *hupL*, and hypothetical proteins Tery_1886 and Tery_0848, while the most significantly downregulated genes were the Fe stress gene *isiA* (*Tery_1667*) and its homologs (*isiA*-like, *Tery_2483/Tery_2484/Tery_2485*), a gene cluster of putative transcriptional regulators (*Tery_1663/1664/1665*), and genes encoding fructose-1,6-bisphosphate aldolase (*fbaAI*), phycobilisome linker polypeptide *cpcG2* (*Tery_2486*), and hypothetical protein Tery_1686 (Fig. S3 and S4). Compared with the LFe control, transcript levels decreased by 70% and 99% for *idiA* and *isiA* genes, respectively, in response to the HFe treatment (Table S2), which was consistent with the qPCR result (Fig. 1).

### Expression of genes for Fe acquisition and homeostasis

**Fig 3:**
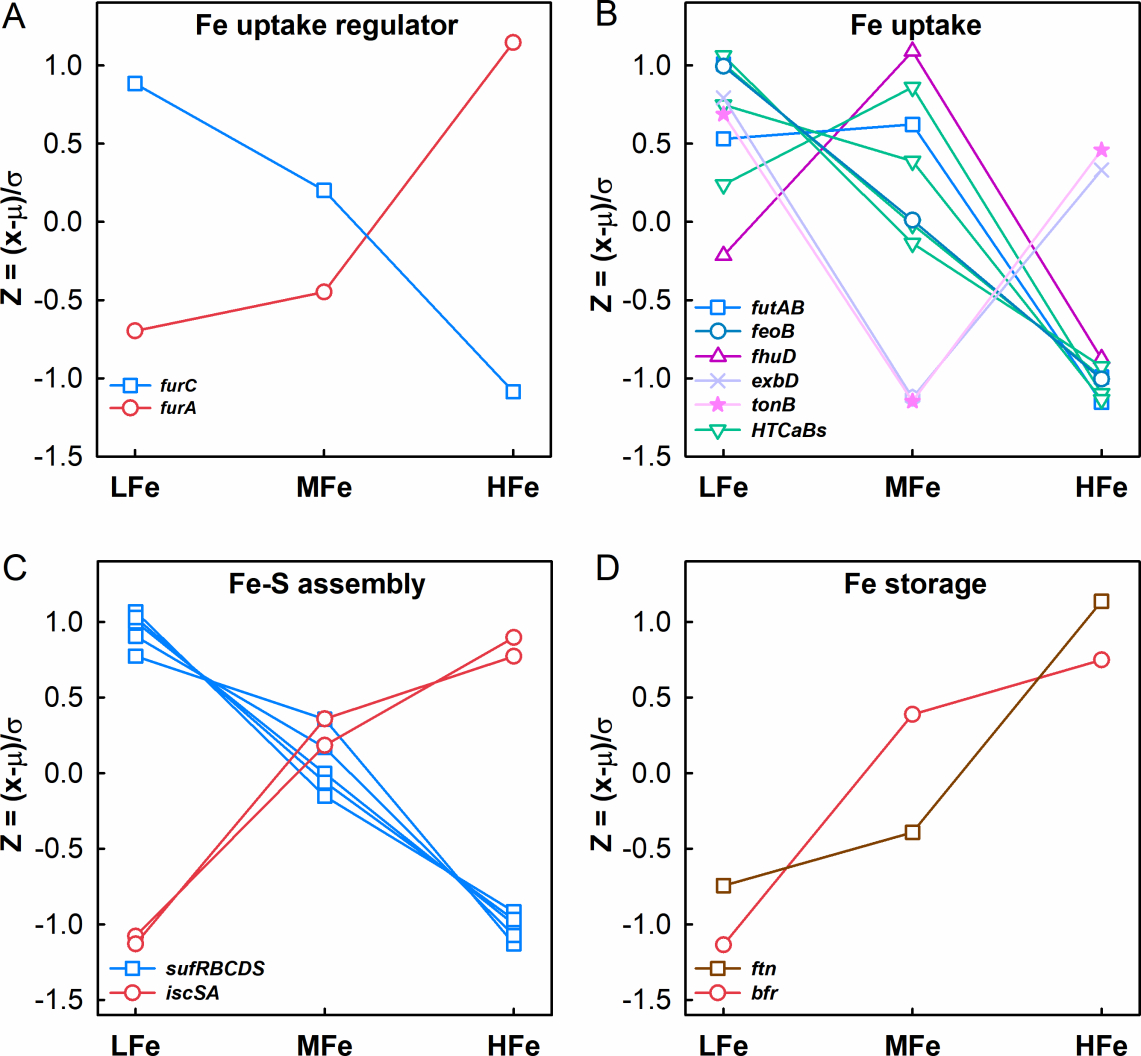
Changes in the expression of genes related to iron acquisition and utilization in *T. erythraeum* IMS101 upon iron supplementation. Shown are genes for Fe homeostasis regulatory factors (**A**), Fe absorption and acquisition (**B**), Fe-S cluster assembly (**C**), and Fe storage (**D**). The relative abundance of gene transcripts at each Fe supplementation concentration was log_2_ transformed and plotted according to the formula *Z* = (*x −* μ)/σ, where *x* is the mean of log_2_CPM in one treatment, μ is the mean of log_2_CPM values in all treatments, and σ is the standard deviation of the overall normal distribution curve. This formula centers and scales a variable to mean 0 and standard deviation 1, which ensures that the criterion for finding linear combinations of the predictors is based on how much variation they explain and, therefore, improves the numerical stability.

The transcript level of *furA* encoding the global transcriptional regulator of intracellular Fe homeostasis increased twofold, whereas that of the other Fe homeostasis regulator gene *furC* decreased by 86% in the HFe treatment relative to the LFe control (Fig. 3A). Transcription of the genes for ferric (*futA*/*idiA*, *futBC*) and ferrous (*feoAB*) Fe transport was also significantly downregulated. Transcript levels of genes coding for proteins with hemolysin-type calcium-binding (HTCaB)-like domains putatively involved in extracellular particulate Fe absorption (*Tery_0419*, *Tery_0424*, *Tery_2055*, and *Tery_2710*), and siderophore absorption system (*fhuD_3943, exbD_4449, tonB_2593*) (46, 47, 53) decreased upon the HFe treatment (Fig. 3B; Table S1). The two sets of Fe-S cluster assembly genes, i.e., the gene *iscS* and *iscA* and the *suf* operon *sufRBCDS*, showed opposite expression patterns, with *iscS* and *iscA* upregulated while *sufRBCDS* downregulated (Fig. 3C). To our expectation, the expression of two Fe storage genes, *ftn* encoding ferritin and *bfr* encoding heme-containing bacterioferritin, were both upregulated (Fig. 3D).

### Expression of genes involved in carbon and nitrogen cycling

**Fig 4:**
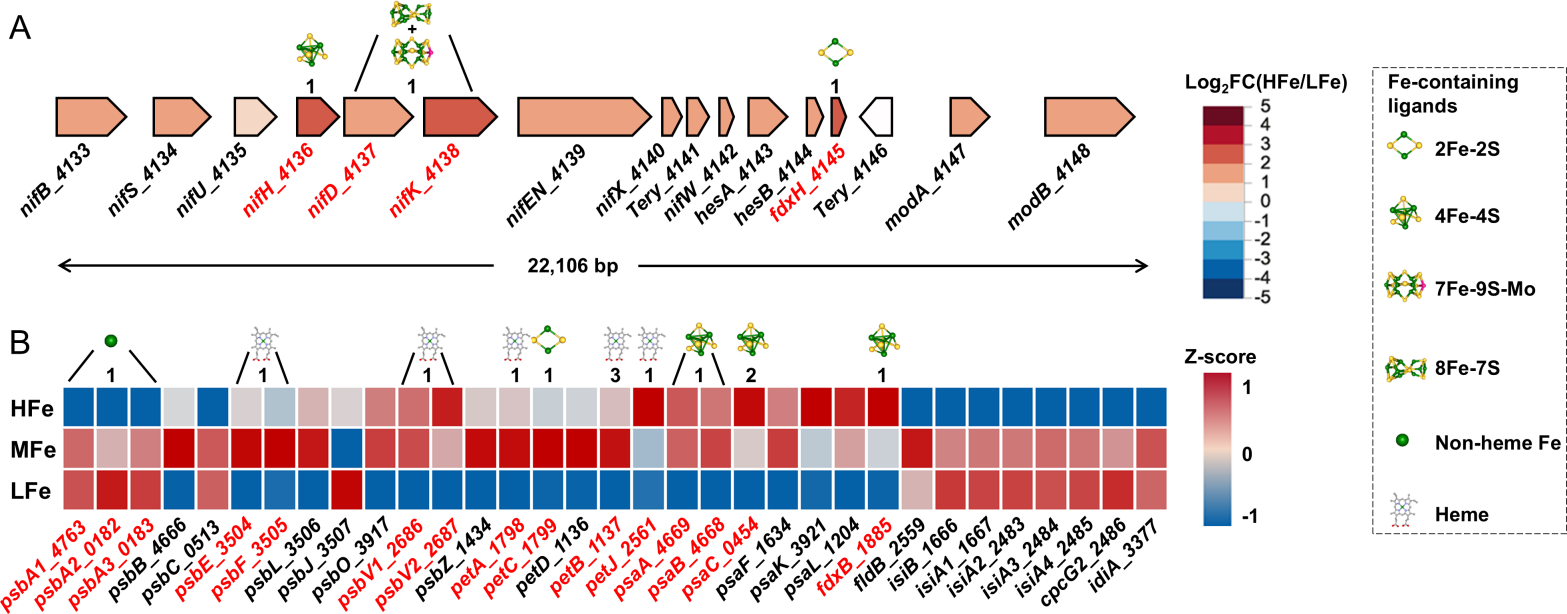
Photosynthetic and N_2_-fixing genes differentially expressed upon iron supplementation. (**A**) Expression of genes involved in N_2_ fixation is shown as fold changes in the level of transcripts between the HFe and LFe treatments. Also shown is the organization of genes in the 22,106 bp long *nif* gene cluster. (**B**) Expression of genes involved in photosynthesis is shown as *Z*-scores corresponding to each iron supplementation treatment. *Z*-scores were calculated according to the following formula: *Z* = (*x −* μ)/σ, where *x* is the mean of log_2_CPM in one treatment, μ is the mean of log_2_CPM values in all treatments, and σ is the standard deviation of the overall normal distribution curve. Genes are labeled with gene symbol (if available) followed by locus tag of the open reading frame (e.g., *psbA1_4763* denoting the *psbA1* gene in the locus tag *Tery_4763* in the *Trichodesmium* genome). For the gene products associated with different forms of Fe (in red text), the numbers and types of the corresponding Fe ions/compounds are given. All Fe-containing molecules are shown in ball-and-stick representation with the Fe, S, Mo atoms depicted as green, yellow, magenta spheres, respectively.

With the increase of supplemented Fe, almost all genes involved in N_2_ fixation were upregulated (Fig. 4A), particularly those having a high iron demand, such as the *nifHDK* gene cluster. The *modAB* genes involved in the molybdate transport and the *hupSL* genes encoding H_2_ uptake hydrogenase were also upregulated under the HFe treatment. On the contrary, the ammonia (*amt*), nitrate/nitrite (*nrt*), and urea (*urtABCDE*) transport genes, as well as the nitrate (*narB*) and nitrite (*nirA*) reductase genes were downregulated (Table S1).

**Fig 5:**
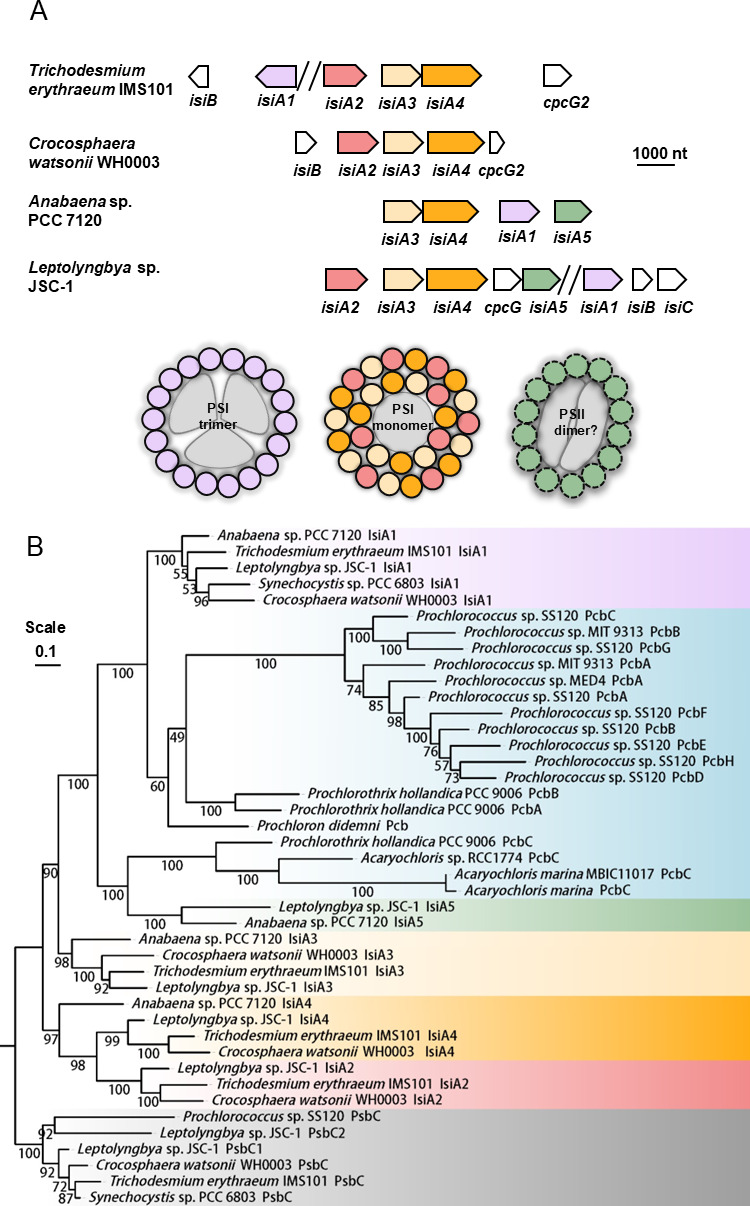
Diversity and conservation of *isiA* gene family in cyanobacteria. (**A**) Variants of *isiA* genes in *Trichodesmium erythraeum* IMS101, *Crocosphaera watsonii* WH0003, *Anabaena* sp. PCC7120 and *Leptolyngbya* sp. JSC-1 genomes, and their possible roles in the formation of super-complexes around photosystems under iron limitation conditions. Discontinued regions are marked with “//.” (**B**) Maximum likelihood tree showing the phylogenetic relationship of *isiA*, *psbC,* and *pcb* genes found in diverse cyanobacteria. The phylogenic tree was constructed with IQ-TREE using aligned amino acid sequences of the IsiA homologs and was rooted with PsbC as the outgroup. Numbers at the branch denote the topological robustness of the tree evaluated by Bayesian criterion with 1,000 bootstrap replicates. Tree scale represents amino acid substitutions per site.

In addition, the expression of genes coding for phycobilisome, PSII and PSI proteins, cyt *c*_6_, ferredoxin-NADP^+^ reductase, NAD(P)H dehydrogenase, cytochrome oxidase, ATP synthase, CO_2_ concentrating enzymes, Calvin cycle enzymes, tricarboxylic acid cycle enzymes, and the ferredoxin-thioredoxin enzymes including ferredoxin-thioredoxin reductase (FTR), ferredoxin (FdxH/B), and thioredoxin (Trx) was also upregulated under Fe-replete conditions (Table S1). However, transcript levels of some genes decreased upon the HFe treatment. For example, the three copies of *psbA* gene encoding D1 protein, *psbA1* (*Tery_4763*), *psbA2* (*Tery_0182*), and *psbA3* (*Tery_0183*), were downregulated by 1.4, 3.5, and 2 times, respectively (Fig. 4B; Table S1). In addition, *fbaAI* encoding fructose-1,6-bisphosphate aldolase, *isiB* encoding flavodoxin, and *cpcG2* encoding the phycobilisome rod core linker protein were downregulated 74, 4, and 30 times, respectively. As expected, the iron stress-inducible gene *isiA* (*Tery_1667*) was downregulated about 100 times upon the HFe treatment. However, unexpectedly, the other three *isiA*-like gene copies (*Tery_2483*, *Tery_2484,* and *Tery_2485*) (Fig. 2 and 5) exhibited an over 300 times further lower transcript level, the largest change in gene expression among all the DEGs (Fig. 4B; Table S2).

### Expression of genes involved in chlorophyll *a*, heme, and vitamin B_12_ biosynthesis

**Fig 6:**
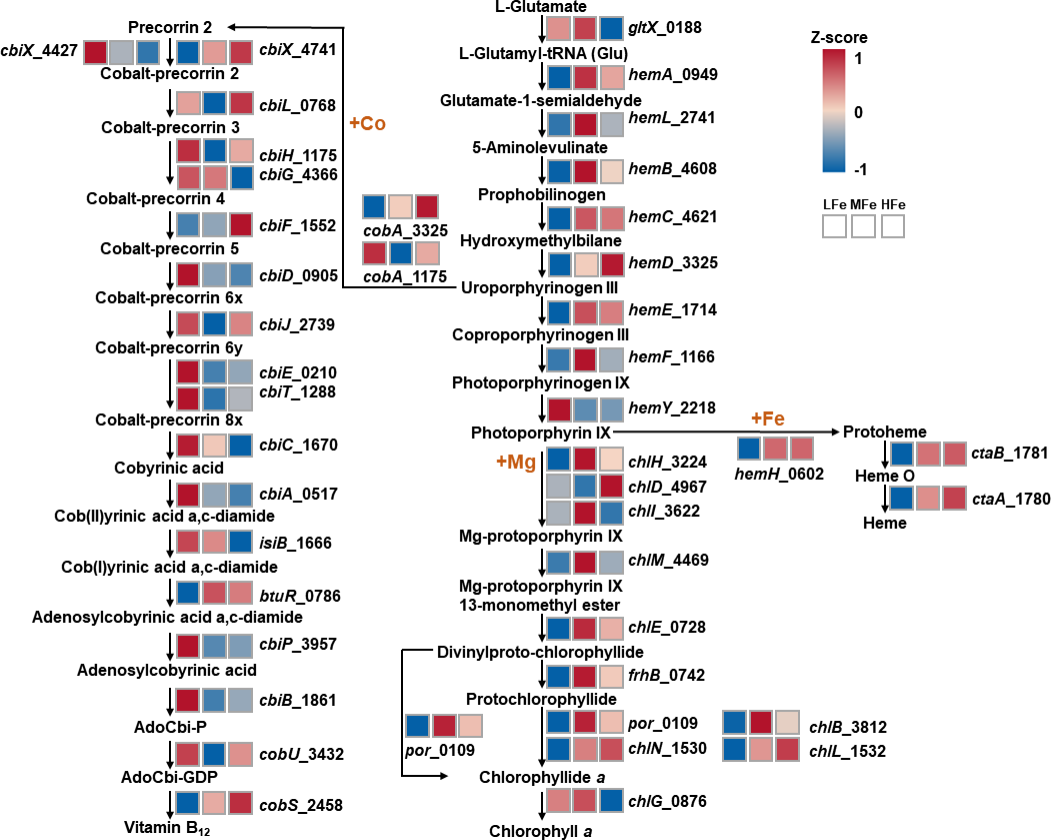
Expression of genes involved in the *de novo* synthesis of chlorophyll *a*, vitamin B_12_, and heme upon iron supplementation. Most genes in the biosynthesis pathways of chlorophyll *a* and heme were significantly upregulated under the HFe treatment, while genes in the vitamin B_12_ biosynthesis pathway were significantly downregulated. Expression of genes is shown as *Z*-scores corresponding to each iron supplementation treatment. *Z*-scores were calculated according to the following formula: *z* = (*x −* μ)/σ, where *x* is the mean of log_2_CPM in one treatment, μ is the mean of log_2_CPM values in all treatments, and σ is the standard deviation of the overall normal distribution curve. The genes are labeled with a gene symbol (if available) followed by a locus tag of the open reading frame (e.g., *chlM_4469* denoting the *chlM* gene in the locus tag *Tery_4469* in the *Trichodesmium* genome).

**Fig 7:**
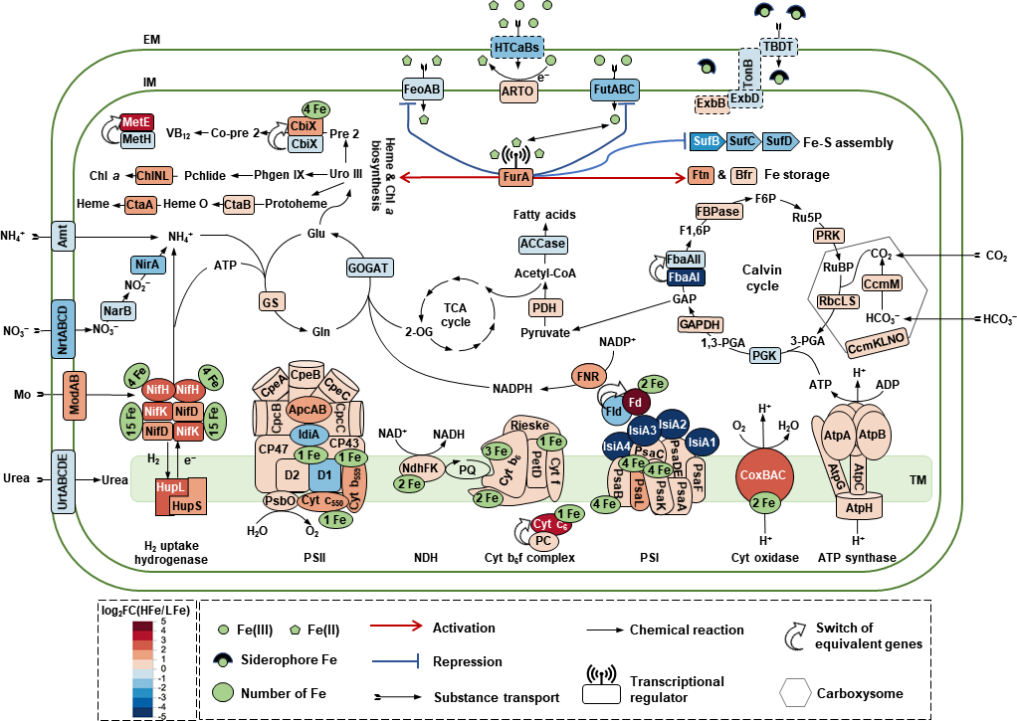
Schematic diagram illustrating significantly regulated gene products for coordinated nutrient transport and carbon and nitrogen metabolism in *T. erythraeum* IMS101 in acclimation to increased Fe availability. Fe supplementation promotes the expression of the genes encoding major Fe-binding metalloproteins involved in photosynthetic electron transport and structural subunits of the nitrogenase complex. The upregulated photosynthesis and N_2_ fixation are accompanied by a balance between the central carbon and nitrogen metabolism, which is tightly linked through the GS-GOGAT cycle. The Fe redox is primarily under the control of the ferric uptake regulator FurA that functions as a transcriptional repressor of genes for Fe utilization, specifically those encoding the transporters of extracellular particulate (HTCaBs) and siderophore (FhuD) Fe, the plasma membrane transporters of ferric (FutABC) and ferrous (FeoAB) Fe, and the Fe-S cluster assembly proteins (SufBCDS). FurA also, possibly indirectly, functions as an activator in controlling the expression of genes encoding Fe storage ferritins (Ftn and Bfr) and proteins involved in heme and chlorophyll *a* biosynthesis. Moreover, the transcription of genes for nitrogen transport and utilization (*amt*, *nrtABCD*, *urtABCDE*, *narB*, and *nirA*) is likely under the control of a sophisticated network involving the Fur family. Proteins putatively involved in the extracellular particulate Fe-siderophore adsorption are boxed in dotted lines. Abbreviations are used as follows: TCA, tricarboxylic acid; Glu, glutamate; Gln, glutamine; GS, glutamine synthetase; GOGAT, glutamate synthase; Uro III, uroporphyrinogen III; Phgen IX, photoporphyrinogen IX; Chl *a*, chlorophyll *a*; Pchlide, Protochlorophyllide; Pre 2, precorrin 2; Co-pre 2, cobalt-precorrin 2; RuBP, ribulose-1,5-bisphosphate; 3-PGA, 3-phosphoglycerate; 1,3-PGA, 1,3-diphosphoglycerate; F1,6P, fructose-1,6-bisphosphate; F6P, fructose-6-phosphate; Ru5P, ribulose 5-phosphate; 2-OG, 2-oxoglutarate; EM, extracellular membrane; IM, intracellular membrane; TM, thylakoid membrane.

Upon the HFe treatment, the expression of genes involved in heme *de novo* synthesis was upregulated by 2–3 times, while that of genes for vitamin B_12_ synthesis was downregulated by 60%–80% (Fig. 6). The two *cbiX* genes encoding cobaltochelatase also exhibited opposite expression patterns; *Tery_4741* was upregulated by 2 times, while *Tery_4427* was downregulated. So did the expression of the two genes encoding methionine synthase, with *metE* upregulated by 10 times, while *metH* downregulated (Fig. 7).

## DISCUSSION

Our measurements of physiological parameters such as growth, photochemical efficiency, and N_2_ fixation, coupled with the transcriptome sequencing analysis, unveiled the molecular mechanisms, whereby IMS101 acclimatizes to fluctuations in Fe availability. The strategic remodeling of gene expression in IMS101 mainly includes (i) upregulating Fe-demanding metabolic genes under Fe-replete conditions, (ii) enhancing light-harvesting capacity through upregulation of multiple iron stress genes under Fe-deplete conditions, (iii) substitutive expression of functionally equivalent genes depending on Fe availability, and (iv) mobilizing transcriptional regulators for the global Fe homeostasis and metabolic C/N balance.

### Transcriptional upregulation of key metabolic genes in response to increased Fe availability

The three distinct metal prosthetic groups of nitrogenase alone require 19 Fe atoms (62). In IMS101 under Fe limitation, N_2_ fixation is primarily downregulated in order to maintain the normal functions of other metabolic pathways (14, 47). Accordingly, with the increase in supplemented Fe in the culture media, the expression of almost all genes for N_2_ fixation was upregulated, leading to enhanced nitrogenase activity (Fig. 1) and the production of ammonia (NH_3_) and hydrogen (H_2_). Transcription of the *hupSL* (*Tery_3369/3368*) gene cluster encoding the small and large subunits of the H_2_ uptake hydrogenase (63) was upregulated under the HFe treatment (Table S2). The enhanced *hupSL* transcription can facilitate the reuse of H_2_ by the H_2_ uptake hydrogenase to regenerate electrons for various cell functions, as well as to produce ATP through oxyhydrogen reaction to minimize energy loss and O_2_ damage to nitrogenase (64, 65). Moreover, transcription was also upregulated upon the HFe treatment for the key photosynthetic genes, including those encoding the PSII reaction center proteins (*psbB/E/F/L/O/V/Z*), cytochrome *b*_6_*f* complex (*petCA, petBD*), PSI reaction center proteins (*psaA/B/C/F/L*), as well as the light-harvesting phycobilisomes (*cpcB*, *cpeB*, *apcA*) (Fig. 4 and 7). The presence of Fe-containing ligands is crucial for sustaining the structural integrity and proper functioning of the photosynthetic pathway. Indeed, Fe deprivation-induced transcriptional repression of these genes may restructure the photosynthetic pathway, leading to an increase in the PSII:PSI ratio (53) or conservation of intracellular Fe resources by altering ATP production pathways (e.g., via a short water-water cycle from PSII to a midstream oxidase or through electron transport from PSII to the respiratory terminal oxidase) (52).

Furthermore, the expression of genes encoding FTR (Tery_1567), Fdx (Tery_4145, Tery_1885), and Trx (Tery_1699) was upregulated under the HFe condition (Table S2). These proteins constitute the ferredoxin-thioredoxin system that converts the reducing power generated from light-activated electron transport in the thylakoid membrane into regulatory thiol signals in the stroma and hence impact the activities of several Calvin cycle enzymes (66). Although Fe is not required for enzymes involved in the Calvin cycle (52), the ensuing upregulation of these genes following the HFe treatment is probably a relief from the Fe stress in promoting the activities of Calvin cycle enzymes.

### Enhancing light-harvesting capacity through the expansion of the *isiA* gene family

Intriguingly, our study revealed an *isiA*-like gene cluster *Tery_2483/2484/2485* (67) with the greatest expression change between the LFe and HFe treatments. This cluster is strikingly similar in gene sequence and synteny (Fig. 5A) to the *isiA2-isiA3-isiA4-cpcG2-isiA5* gene cluster in the freshwater cyanobacterium *Leptolyngbya* JSC-1, which was upregulated approximately 500 times under Fe limitation (68). While these additional IsiA-like copies are phylogenetically closely related to the *psbC*-encoded PSII reaction center core subunit CP43 (Fig. 5B), which is constitutively expressed (69, 70), they all lack the large loop on the lumenal side that is typical for CP43 (Fig. S5). It is, therefore, legitimate to clarify *Tery_2483*, *Tery_2484,* and *Tery_2485* as homologs (*isiA2*, *isiA3,* and *isiA4*) of the *isiA* gene (hereafter *isiA1*, *Tery_1667*), rather than *psbC* as currently annotated in the IMS101 genome. The PsaL domain at C-terminus of IsiA4 is suited to the formation of IsiA loops around PSI monomer while inhibiting PSI oligomerization (68, 71). Transmission electron microscopic analysis suggests that IsiA1 may be involved in the formation of super-complex with trimeric PSI, while IsiA2/A3/A4 with monomeric PSI (68, 71). As a result, the number rises from a single ring of 17–18 IsiA copies surrounding PSI trimer (72) to double rings of 14–35 IsiA copies surrounding PSI monomer (73), which greatly enhances the light harvesting and energy transferring efficiency of the monomeric PSI-IsiA super-complex (68, 73).

The relative transcript change of *isiA2-isiA3-isiA4* was two times higher than that of *isiA1* (Table S2), suggesting that *isiA2-isiA3-isiA4* is more sensitive to Fe stress. Phylogenetic analysis showed that IsiA2, IsiA3, and IsiA4 each forms a monophyletic clade that is clearly distinct from IsiA1, implying that the expansion of IsiA2/A3/A4 may have occurred independent of IsiA1, and likely emerged much later (e.g., after the great oxidation event) to facilitate acclimation to the worsening Fe limitation at geological timescales (Fig. 5B). This array of multiple *isiA* genes also exists in filamentous, heterocystous *Anabaena* sp. PCC 7120 and some of the unicellular marine diazotrophs such as *Crocosphaera* of large cell size (e.g., WH0003), but not small cell size (e.g., WH8501) (Fig. 5A). Under Fe-deficient conditions, cyanobacteria with multiple *isiA* genes have the advantage to accumulate IsiA products (71), resulting in reduced cellular content of the PSI reaction center and reduced levels of transcription of PSI core genes. Increased copies of *isiA* also contribute to higher photosynthetic efficiency in larger *Crocosphaera* under Fe limitation (74) and may be beneficial for low-light adapted *Synechococcus* ecotypes (75). Similarly, it is reasonable to speculate that *Trichodesmium* with multiple copies of *isiA* may exhibit superior performance in both light capture and photoprotection compared to those without.

### Flexibility in substitutive gene expressions depending on Fe availability

The flexibility of cyanobacteria in acclimation to various environmental conditions can be achieved through the substitutive expression of different gene copies or genes encoding functionally equivalent proteins. In the former case, for example, Clarke et al. (76) found that in *Synechococcus* sp. PCC 7942, two different forms of D1 proteins (called D1:1 vs D1:2) vary in abundance between normal and high-irradiance photoinhibition conditions, as inferred from different patterns in the expression of their respective genes (i.e., *psbA1* for D1:1 and *psbA2*/3 for D1:2) (76). This regulation of gene expression of different *psbA* copies is executed so that *de novo* synthesis of D1 protein can be strictly controlled to maintain the stability of the photosynthetic apparatus under normal conditions or under environmental stress with a rapid turnover of D1 proteins (77). This pattern of *psbA* gene expression appeared to exist in IMS101 subjected to different concentrations of Fe supplemented (Table S1). Another example is the switch in expression between two copies of the gene encoding fructose-1,6-bisphosphate aldolase (FBA) in the Calvin cycle in IMS101 under Fe-replete conditions (Fig. 7; Table S1), where the expression of *Tery_1687* (*fbaAI*) encoding class I FBA (FbaAI) was replaced with that of *Tery_4099* (*fbaAII*) encoding Fe^2+^-dependent class II FBA (FbaAII). The ratios of *fbaAI/fbaAII* transcription were 0.03 and 2.03 under the HFe and LFe treatments, respectively. This finding is consistent with those reported in diatoms (51, 78). Actually, because of the significant changes in the relative abundance of FbaAI and FbaAII under fluctuating Fe concentrations, the ratio of FbaAI/FbaAII is often used as an indicator for Fe availability (46, 47, 54). In addition, under the Fe-limited conditions, genes encoding Cu-containing plastocyanin (PC; Tery_2563) and flavodoxin (IsiB; Tery_1666) were upregulated to substitute Fe-containing cytochrome *c*_6_ (Cyt *c*_6_; Tery_2561) and ferredoxin (Fdx; Tery_4145), respectively. PC is crucial for phytoplankton in the open oceans where Cu is relatively abundant but Fe is scarce, and the replacement of Cyt *c*_6_ by PC reduces the Fe requirement by roughly 10%, while the electron transport rates are still maintained at a high level (79).

There is also a trade-off in the synthesis of porphyrin compounds. Chl *a*, heme, and vitamin B_12_ are porphyrin-derived compounds with Mg, Fe, and Co as a ligand, respectively. Since the synthesis of chlorophyll and heme depends on Fe as a cofactor (80, 81), Fe deficiency in IMS101 may reduce the accumulation of chlorophyll and heme, causing more porphyrin precursors to be diverted to the vitamin B12 synthesis pathway by enhancing the absorption of Co (Fig. 6). A similarly negative relationship between Co concentrations in solution and intracellular Fe quota has been observed in *Microcystis aeruginosa* (82). Moreover, the increase in synthesis of vitamin B_12_ under LFe also activates the B_12_-dependent methionine synthase (MetH; Tery_2492) to replace B_12_-independent methionine synthase (MetE, Tery_0847) (Fig. 6 and 7). These results were consistent with the proteomics-based observation that MetE was more abundant under low B_12_ availability in the diatom *Phaeodactylum tricornutum* (83). It has also been found that phytoplankton preferentially uses MetH to save intracellular resources under environmental stresses such as Fe limitation, as the expression of MetE will lead to a large consumption of resources and energy because the catalytic activity of MetE was about 100 times lower than that of MetH (1, 84). Furthermore, there are two copies of the *cbiX* gene in IMS101 encoding cobaltochelatase, which catalyzes cobalt insertion in vitamin B_12_ biosynthesis. Under high Fe concentrations, transcription of *cbiX1* (*Tery_4741*), which encodes a [4Fe-4S]-containing protein, was significantly upregulated compared to that of *cbiX2* (*Tery_4427*), whose gene product does not require the [4Fe-4S] ligand (85).

### Global regulation for Fe homeostasis

In analogy to what has been established in other cyanobacteria, the regulator Fur family is involved in the global regulation of gene expression to maintain Fe homeostasis (86). The Fur family proteins include FurA, FurB, and FurC, which participate in Fe transport, biosynthesis, and cellular redox balance progresses. All the three Fur protein homologs exist in IMS101 (Fig. S6). Among them, FurC (PerR, Tery_3404) was found to be a metal-dependent H_2_O_2_ sensor (87), and the downregulation of *furC* upon the HFe treatment in our data indicated that the oxidative stress was relieved by Fe supplementation (Fig. 3A).

In IMS101 under Fe-replete conditions, the transcription of ferric uptake regulator FurA (Tery_1958) was induced, leading to tighter repression of Fe acquisition genes including the *feoAB* and *futABC* operons for transport of Fe^2+^ and Fe^3+^, respectively (Fig. 7). Note that *feoAB* was consistently downregulated under both the MFe and HFe concentrations, whereas *futAB* was only downregulated upon the HFe treatment (Fig. 3B), suggesting that *Trichodesmium* cells prioritize Fe^2+^ absorption. Moreover, the expression of genes coding for Fe storage ferritins Ftn (Tery_4282) and Bfr (Tery_2787) was increased (Fig. 3D). These changes implied the role of FurA in controlling the expression of genes for Fe transport and storage to balance free Fe ion concentrations, which was also previously analyzed in the cyanobacteria *Synechocystis* sp. PCC 6803 (88) and *Anabaena* sp. PCC 7120 (89).

In addition, FurA could also be involved in the regulation of genes involved in the *de novo* synthesis of Fe-containing compounds such as the Fe-S clusters and hemes (Fig. 7) (90, 91). Three sets of genes, the *sufBCDS* (*Tery_4355/6/7/8*) gene cluster*, the nifU* (*Tery_4135*) *and nifS* (*Tery_4134*) genes, and the *iscS* (*Tery_4044*) and *iscA* (*Tery_3951*) genes are involved in Fe-S biosynthesis in *Trichodesmium* (90). The alternative regulation of these Fe-S assembly genes under varied Fe concentrations suggests the flexibility of *Trichodesmium* in acclimation to Fe availability. The *iscS* and *iscA* gene products are involved in general Fe-S cluster assembly (90), whereas *nifU* and *nifS* gene products only participate in the assembly of nitrogenase-specific Fe-S cluster (92, 93). These two systems were upregulated in IMS101 under the HFe treatment, possibly to meet the increasing demand for Fe-S cluster in fast-growing cells (Table S2). However, *sufBCDS* is usually induced by oxidative stress and Fe-restricted conditions to protect cells from ROS damage (94). In *Synechocystis*, the expression of the *sufBCDS* gene cluster is under the control of the transcriptional repressor SufR (95), an oxidative stress sensor that contains a [4Fe-4S] ligand for sensing the intracellular Fe level (96). Under Fe depletion, SufR loses its capacity for repression because the [4Fe-4S] ligand becomes scarce and the *sufBCDS* gene cluster would become highly expressed. In many bacteria, this conflict is solved by an sRNA regulator that is only transcribed under Fe starvation due to released repression from a FurA-controlled promoter (97, 98). For example, in *Synechocystis*, the sRNA IsaR1 post-transcriptionally represses *sufBCDS* (and several other genes) under the Fe limitation condition (98). Because a similar regulatory effect on the *sufBCDS* expression was not detected in this study (Fig. 7), such sRNAs may not exist in IMS101, pointing at a potentially remarkable difference in the regulatory machinery controlling the acclimation to low iron.

Another group of genes likely directly targeted by FurA are the iron stress protein-encoding gene *isiA1* (*Tery_1667*) and the flavodoxin-encoding *isiB* (*Tery_1666*), which were co-transcriptionally upregulated by 100 times and 4 times, respectively, under Fe limitation (Fig. 4B; Table S2). In *Synechocystis*, Fur binds to the Fur box situated upstream of the *isiAB* operon, thus preventing the binding of RNA polymerase and inhibiting the transcription of *isiAB* under Fe-replete conditions (88, 99, 100). Putative Fur boxes for nine Fe-responsive genes (*isiAB*, *ftnA*, *feoA*, *hemD*, *hemH*, *futA*, *futC*, *ctaA*, and *bfr*) were identified in IMS101, and the sequences of these Fur binding sites are extremely conserved (Fig. S7). It is hence a strong evidence that FurA directly or indirectly regulates the transcription of these genes given the status of Fe availability (97).

It is worth noting that, due to limited annotations of the IMS101 genome and a high number of genes of unknown functions, the roles of many DEGs are unclear. For example, the *Tery_1663/1664/1665* gene cluster, encoding a putative Crp/Fnr family transcriptional regulator, a hypothetical protein, and a hydrolase (Fig. 2), was upregulated more than 100-fold at LFe (Table S2), but its regulatory mechanism is entirely uncharacterized. Vice versa, the four small heat shock protein genes (*hsp20*, *Tery_3061/3062/3063/3064*) were upregulated more than 10-fold under the HFe treatment (Fig. S3; Table S2), but their function in acclimation to Fe availability has not been experimentally addressed. One speculation is that under the nutritionally adequate conditions these chaperones are needed to bring the additionally synthesized protein into the correct conformation. What is even more intriguing is the cryptic polyketide synthase (PKS)-encoding gene cluster *Tery_3819/3820/3821/3822*, which was upregulated significantly under the LFe treatment (Fig. 2). Recent studies in *Escherichia coli* have shown the involvement of the pks island in synthesizing secondary metabolites with antimicrobial activity (101), some of which are implicated in prophage induction (102). *Trichodesmium* colonies are usually symbiotic with heterotrophic epibionts and other microorganisms including diatoms, proteobacteria, and other cyanobacteria (103–106). As the IMS101 culture was not stringently axenic in this study, some symbionts may assist the Fe absorption and storage of *Trichodesmium* under the HFe condition, while there is a competition between them for Fe utilization under the LFe condition. It is tempting to speculate that low Fe supply may result in increased synthesis of these antimicrobial metabolites, thereby favoring *Trichodesmium* colonization in interbacterial competition. A better characterization of these genes could potentially improve our understanding of their involvement in the cellular regulatory networks. In addition, the data set generated in this study can be further analyzed using other approaches, such as electron microscopic visualization of the IsiA-PSI/PSII super-complexes (68), epigenetic (107), or the proteomic (108) analysis of the DEGs. Meanwhile, protein modifications, such as acetylation and recombination in binding coenzymes and prosthetic groups, will influence enzymatic activity. Thus, a comprehensive analysis of transcriptomics, proteomics, metabolomics, and epigenomics might provide novel insight into *Trichodesmium* acclimation to Fe availability.

### Conclusions

In this study, by subjecting *Trichodesmium erythraeum* IMS101 to increasing concentrations of supplemented iron (Fe), we observed enhanced growth, photosynthesis and N_2_ fixation, and a concomitant remodeled transcriptome underpinning the physiological response of *T. erythraeum* IMS101 to increased Fe availability. Our data demonstrate the regulation of the Fe hemostasis by the transcriptional regulator FurA in controlling the expression of genes for Fe transport (e.g., *futABC* and *feoAB*), Fe storage as ferritin (*ftn*), and Fe-S assembly (*sufRBCDS*). Transcriptome profiling further unveils the plasticity in the transition of cellular processes for Fe resource allocation, for example, the substitutive switch between functionally equivalent genes and the trade-off between heme/Chl *a* and B_12_ biosynthesis depending on Fe availability. Moreover, the identification of the *isiA2/A3/A4* gene cluster expands our recognition of the iron stress gene *isiA*, suggesting that this gene family may have a more dynamic/plastic role than previously thought. Harboring multiple *isiA* copies, particularly in conjunction with genes for antimicrobial metabolites synthesis, may facilitate *Trichodesmium* to cope with more severe Fe deficiency to colonize the natural habitats. Furthermore, our study provides broader genomic insight into the fundamental cellular processes responsible for acclimation to Fe availability in a representative, prominent marine diazotroph, given that IMS101 is frequently considered a model organism exhibiting unique characteristics. The identification of differentially expressed genes enables the versatility and flexibility in choice of indicator genes for monitoring fluctuations of Fe in the environment. Last but not least, our findings offer important implications for future studies on whether the adaptation mechanisms described here are applicable to other diazotrophs and marine bacteria in general, which is crucial for understanding their response to Fe limitation and how they persist into the future in an era of global climate change.

## MATERIALS AND METHODS

### Culture conditions and experimental design

*Trichodesmium erythraeum* strain IMS101 (hereafter IMS101) was obtained from the Provasoli-Guillard National Center for Marine Algae and Microbiota at Bigelow Laboratory (East Boothbay, Maine, USA) and grown in Aquil-tricho medium (109) prepared using surface natural seawater collected from the South China Sea at the South East Asia Time-series Study (SEATS, 18°N and 116°E) station. The bulk seawater was first filtered through a 2 µm pore size, 142 mm diameter polycarbonate filter membrane (Millipore, Bedford, MA, USA) to remove impurities, then autoclaved at 121°C for 20 min, and supplemented with NaHCO_3_, KH_2_PO_4_, vitamins, and trace metals following the medium recipe. The medium was aliquoted into 2 L transparent polycarbonate bottles (Nalgene, Rochester, NY, USA), which were soaked in 1 N HCl overnight and thoroughly rinsed three times with ultrapure Milli-Q water before autoclaving.

IMS101 was cultivated at 26°C and 120 µmol quanta m^−2^ s^−1^ illumination under a 14 h:10 h light-dark cycle in 2 L transparent polycarbonate bottles (Nalgene, Rochester, NY, USA). Sterile fresh air was supplied to the culture medium with a syringe filter connected to an air pump. To choose the appropriate Fe supplementation concentrations, preliminary experiments were performed with 1, 10, 50, 100, and 200 nM FeCl_3_ add ed into the prepared medium according to previous studies (56, 110). Based on the observed differences in IMS101 growth, a gradient of 0, 10, and 100 nM FeCl_3_ complexed with 20 µM ethylenediaminetetraacetic acid (EDTA) was finally used to represent the low (LFe), medium (MFe), and high (HFe) Fe supplementation concentrations. To establish steady-state acclimation to differential Fe availability, IMS101 was routinely maintained in each of the LFe, MFe, and HFe media for up to 6 months under a semi-continuous mode (i.e., refreshed with the corresponding medium every 7 days). Cultures freshly diluted to ~1 µg L^−1^ of chlorophyll *a* (Chl *a*) content were used to start a new cycle of the Fe supplementation experiment, and the growth rates of *Trichodesmium* remain stable during the cultivation period (Fig. S1). Biologically triplicate samples (i.e., from different bottles) were collected and used for the following assays. As we cannot complete all the measurements in a single day, we had to sample at different days (but all at the midpoint of the light period). Samples for Chl *a* measurement were collected every other day. The rates of N_2_ fixation, photosynthetic O_2_ evolution, and quantitative PCR (qPCR) assays and transcriptomic sequencing were conducted using samples collected on days 6, 7, and 5, respectively.

### Chl *a* extraction and calculation of specific growth rate

For Chl *a* extraction, 100 mL of algal culture for each Fe supplementation treatment was filtered on a 3 µm pore size, 25 mm diameter polycarbonate filter membrane (Millipore, Bedford, USA). The membrane was then soaked in 90% methyl alcohol and incubated at 65°C for 6 min. After centrifuging at 18,000 × *g* for 10 min, the supernatant was measured at the wavelengths of 650, 665, and 750 nm, and the light absorption values were recorded as *A*_650_, *A*_665_, and *A*_750_, respectively. The Chl *a* concentration was calculated as


$$\mathrm{Chl}a=16.5\times \left(A_{665}-A_{750}\right)-8.3\times \left(A_{650}-A_{750}\right)\times 2\times 1,000/V,$$

where *V* was the volume of the algae, and the Chl *a*-derived specific growth rate (μ) was calculated as


$$\mu =\left[\ln\left(\mathrm{Chl}a_{t}\right)-\ln\left(\mathrm{Chl}a_{t_{0}}\right)\right]/\left(t-t_{0}\right),$$

where Chl *a*_*t*_ and Chl *a*_*t*0_ represent the concentration of Chl *a* at the time of *t* and *t*_0_, respectively.

### Measurement of photosynthetic O_2_ evolution

Photosynthetic efficiency was assessed by measuring the rate of photosynthetic O_2_ evolution using a Clark-type O_2_ microsensor (Unisense, Aarhus, Denmark) suspended in 75 mL of algal cultures. The measurement temperature was set at 25°C and the actinic light intensities were set as 0, 10, 25, 50, 100, 200, 300, 500, 750, 1,000 µmol m^−2^ s^−1^ controlled by the light-emitting diodes. The rate of respiration was measured by keeping the algal cultures in the dark. The photosynthesis-irradiance (P-I) curves were obtained by fitting the data with the hyperbolic tangent model (111). The maximum light utilization coefficient α was given as the light-limited initial slope of the P-I curve, the net maximum photosynthetic rate *P*_max_ (μmol O_2_ mg^−1^ Chl *a* h^−1^) was recorded as the photosynthetic rate under light-saturated conditions, and the saturation irradiance *E_k_* (μmol photons m^−2^ s^−1^) was given as intercept between α and *P*_max_.

### Measurement of N_2_ fixation rate

Samples were collected on day six at the midpoint of the photoperiod for measuring the N_2_-fixation rate via acetylene reduction assay (112). Briefly, 5 mL of algal culture was added into 12 mL headspace vials (Labco, High Wycombe, UK), and 1 mL acetylene gas (>99%) was inserted into the bottle through the septum cap. A bottle of 5 mL pure culture medium was set as a blank. All the bottles were incubated for 2 h under the same condition as algal growth (see above). After the incubation, 100 µL HgCl_2_ was immediately added to the bottles to stop the reaction and fix the samples. Five hundred microliters of gas samples was extracted with a gas injection needle (SGE, Sydney, Australia) and analyzed using a Clarus 580 gas chromatograph (Perkin Elmer, Waltham, MA, USA). The acetylene reduction rate was converted to N_2_-fixation rate using a 4:1 molar ratio and normalized to Chl *a* content.

### Measurement of soluble Fe concentrations

To minimize Fe contamination, trace metal grade ultrapure hydrochloric acid (HCl) (Merck, Germany) and ultrapure Milli-Q water (resistivity ≥ 18.2 MΩ cm) were used to clean the labware. Sampling was performed on an ultraclean workbench. Samples were taken at designated time points during the growth period (0, 2, 4, and 6 days after the inoculation). The IMS101 cultures were filtered on 0.2 µm pore size polycarbonate filter membranes. The filtrates were collected in 50 mL centrifuge tubes and acidified with HCl at a final concentration of 0.02% (wt/vol). Concentrations of dissolved Fe were measured using a custom-built flow analyzer as described by Chen et al. (113). Because EDTA was added in the culture medium, only unchelated, dissolved species of Fe (II + III) (dFe'), i.e., the bioavailable Fe, was detected.

### RNA extraction

Samples for qPCR assays and transcriptomic sequencing were collected by filtering 700 mL of algal culture onto a 3 µm pore size 25 mm diameter polycarbonate membrane (Millipore, Bedford, MA, USA). Total RNA was extracted using the miRNeasy Mini Kit (Qiagen, Hilden, Germany) following the manufacturer’s instructions. Briefly, the cells were lysed with QIAzol reagent, and chloroform was added to the cell homogenate. After centrifugation, the homogenate was further separated into RNA-containing aqueous phase, DNA-containing interphase, and protein-containing organic phases. The aqueous supernatant was precipitated with 1.5 vol ethanol in an RNeasy Mini spin column, and the pellet was washed twice with the RPE buffer included in the kit. Total RNA was eluted by adding RNase-free water to the air-dried spin column and collected into a new collection tube via centrifugation. The content and quality of RNA were assessed using a NanoDrop spectrophotometer (Thermo Fisher Scientific, Waltham, MA, USA) which was calibrated with RNase-free water. Total RNA was treated with the TURBO DNA-free kit containing DNase and removal reagents (Ambion, Austin, TX, USA) following the manufacturer’s protocol. The absence of genomic DNA was confirmed by PCR failing to amplify the 16S rRNA gene using the bacterial universal primers (114). The DNA-free RNA samples were kept at −80°C until further processing.

### Quantitative PCR assay of Fe stress indicator genes

Reverse transcription of RNA samples was conducted using the PrimeScript RT reagent Kit with gDNA Eraser (Takara, Otsu, Japan). The reactions were prepared according to the manufacturer’s instructions, and the reverse transcription steps (15 min at 37°C and 5 s at 85°C) were conducted using a T100 Thermal Cycler (Bio-Rad, Hercules, CA, USA). The TaqMan qPCR was performed according to Shi et al. (14). The cDNA amplification was conducted using the CFX96 Real Time PCR Machine (Bio-Rad, Hercules, CA, USA) with the following thermocycling steps: 50°C for 2 min and 95°C for 10 min at the initial denaturation stage, 95°C for 15 s and 60°C for 1 min at the cycling stage for 40 cycles. Transcript levels of the selected genes were normalized to *rnpB* using the 2^−ΔΔ*C*_*t*_^ method, where *C*_*t*_ represented the number of cycles for genes to reach the threshold (115). Then, Δ*C_t_* values of the HFe and MFe samples were normalized against that of the LFe samples to obtain the relative expression levels of *idiA* and *isiA*.

### Transcriptomic sequencing and bioinformatics analysis

Total RNA samples were shipped on dry ice to Vertis Biotechnologie AG (Freising, Germany), where rRNA was depleted using the Ribo-Zero rRNA Removal Kit (Illumina, San Diego, CA, USA). Then, cDNA libraries were prepared according to the Illumina TruSeq protocol (Illumina, San Diego, USA). The transcriptome sequencing was performed on an Illumina NextSeq 500 platform. One of the replicate samples from the LFe and MFe treatments was not sequenced because of poor RNA extraction (i.e., *n* = 2 for LFe and MFe; *n* = 3 for HFe).

After demultiplexing, adapter sequences and low-quality bases of raw reads in fastq format were trimmed by atropos v1.1.18 (116) with parameters: -e 0.1 -Q 33 -q 20 --trim-n -m 30, and the quality was confirmed using FastQC v0.11.2 (117). PhiX174, sequencing artifacts and human genomic sequences were removed using bbduk.sh and bbmap.sh with parameters: minid = 0.95 maxindel = 3 bwr = 0.16 bw = 12 minhits = 2 qtrim = rl trimq = 10 untrim quick match fast, both scripts are part of the BBTools package v37.24 (available at https://jgi.doe.gov/data-and-tools/bbtools). Ribosomal RNA sequences were removed using SortMeRNA v2.0 (118) with default parameters, and the clean non-rRNA reads were aligned to the IMS101 reference genome using BWA MEM v0.7.12 (119) with default parameters. The number of reads aligned to each gene feature was counted using featureCounts v1.6.0 (120), and differentially expressed genes were called using DESeq2 v1.24.0 (121) with a log_2_ fold change (log_2_FC) cutoff of 1 and an adjusted *P*-value (padj) cutoff of 0.05. The RNA transcript levels were normalized using the default variance stabilizing transformation (vst) method implemented in DESeq2. The trimmed mean of *M*-value (TMM)-normalized read counts in counts per million (CPM) were also calculated using edgeR v3.26.8 (122) to compare gene expression across treatments (Table S1). GO and KEGG functional enrichment analyses were performed using GOstats v2.50 (123) and clusterProfiler v3.12 (124). Statistical analysis was done using R version (3.6.1), and the R script used in this experiment has been uploaded to GitHub at https://github.com/hou-lab/Tricho-Transcriptomics/tree/main.

### Phylogenetic analysis

Amino acid sequences of the IsiA and homologous proteins were aligned using MAFFT version 7.490 (125) with options -auto -maxiterate 1000. Ambiguously aligned regions were removed using trimAl version 1.4 (126) with the gappyout option. Maximum likelihood phylogenetic tree was built using IQ-TREE v2.2.0 (127) based on the best-fitting model automatically detected by ModelFinder (128) implementing the Bayesian information criterion using the -MFP option. Topological robustness of the tree was evaluated by 1,000 ultrafast bootstrap replicates. PsbC sequences were used as the outgroup.

## Data Availability

Raw sequence data for the transcriptome sequencing reported in this study have been deposited in the NCBI Sequence Read Archive under BioProject accession number PRJNA866334.

## SUPPLEMENTAL MATERIAL

Supplemental Figures msystems.01499-24-s0001.docx Figures S1 to S7.

Table S1 msystems.01499-24-s0002.xlsx All processed data of normalized expression.

Table S2 msystems.01499-24-s0003.xlsx List of differentially expressed genes.

ASM does not own the copyrights to Supplemental Material that may be linked to, or accessed through, an article. The authors have granted ASM a non-exclusive, world-wide license to publish the Supplemental Material files. Please contact the corresponding author directly for reuse.
