# Supplementary material for: Transcriptome remodeling drives acclimation to iron availability in the marine N2-fixing cyanobacterium Trichodesmium erythraeum IMS101
Source: mSystems. 2025 Apr 17;10(5):e01499-24. doi: 10.1128/msystems.01499-24 (PMC12090762; doi:10.1128/msystems.01499-24)
Supplement: Supplemental Figures — Figures S1 to S7. [file msystems.01499-24-s0001.docx]

*Supplementary Information of*

**Transcriptome remodeling drives acclimation to iron availability in the marine N_2_-fixing cyanobacterium *Trichodesmium erythraeum* IMS101**

**Xin Zhong et al.**

*Correspondence to*: Tuo Shi (tuoshi@sdu.edu.cn)

**File1: Figures S1–S7 (this file).**

**File 2: All processed data of normalized expression (.xlsx).**

**Table S1.** List of all normalized protein-coding gene expression in *Trichodesmium erythraeum* IMS101 in response to increased iron availability. Relative transcript abundances were represented as log_2_-transformed fold change (log_2_FC) and adjusted p-values (padj) through pairwise comparisons between low (0 nM, LFe), medium (10 nM, MFe) and high (100 nM, HFe) Fe supplementation concentrations (i.e., HFe vs. LFe, MFe vs. LFe, and HFe vs. MFe). For each biological replicate, the normalized gene expression values are shown as read counts in counts per million (CPM). The start/end position and orientation corresponding to each open reading frame (ORF) and gene product annotations are also shown in the table.

**File 3: List of differentially expressed genes in response to increased iron availability (.xlsx).**

**Table S2.** List of differentially expressed genes (DEGs) in response to increased iron availability. A total of 199 DEGs with a log_2_-transformed fold change (log_2_FC) >1 (i.e., |log_2_FC|>1) and an adjusted p-value (padj) <0.05 were identified in response to a gradient from low (0 nM, LFe) to medium (10 nM, MFe) and high (100 nM, HFe) Fe supplementation concentrations through pairwise comparisons (i.e., HFe vs. LFe, MFe vs. LFe, and HFe vs. MFe). Additional Fe-responsive genes whose differential expressions fall below the log_2_FC cutoff are also included. For each biologically replicatd sample, the normalized gene expression values are shown as read counts in counts per million (CPM).





**Figure S1**: Growth of *Trichodesmium* represented as the rates of Chl *a* increase during the semi-continuous cultivation period. The Chl *a*-based specific growth rates were different among the LFe, MFe and HFe treatments (HFe>MFe>LFe), but remain stable per each treatment during the cultivation period.


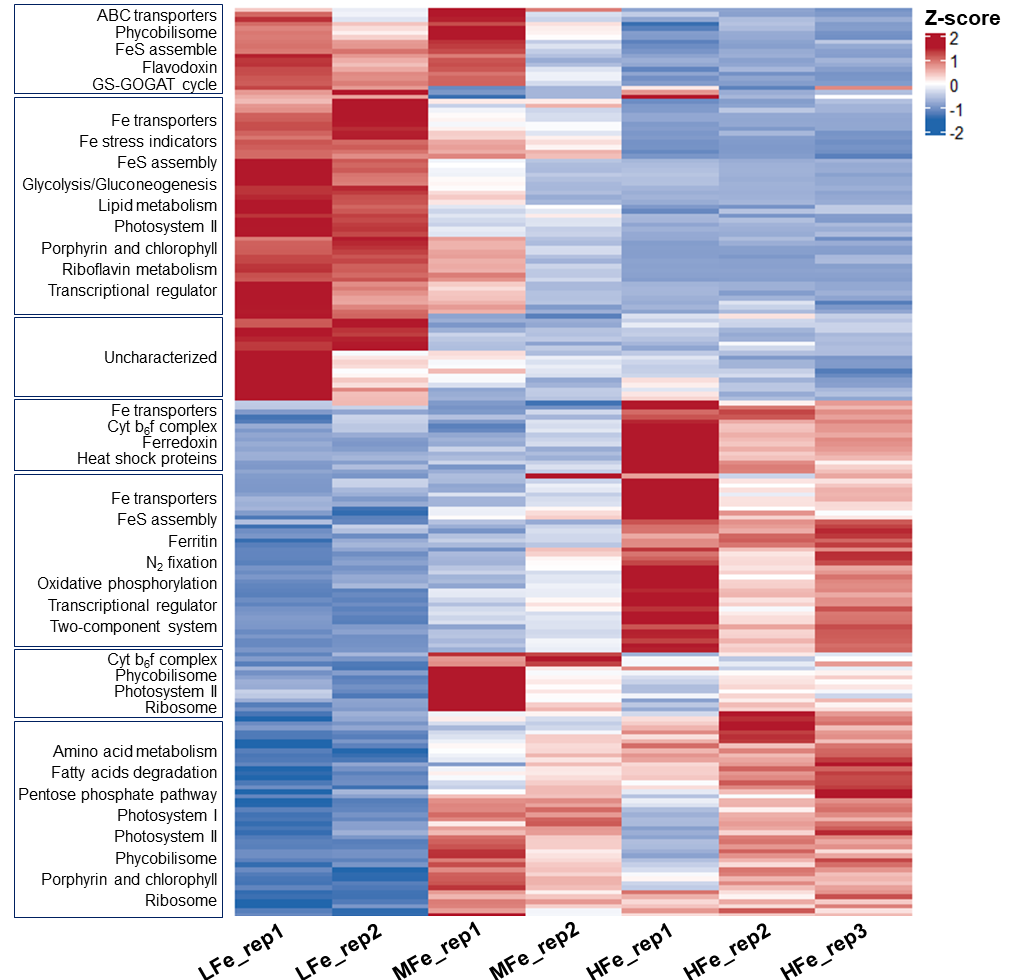


**Figure S2.** Heatmap of the 199 differentially expressed genes (DEGs) in response to a gradient from low (0 nM, LFe) to medium (10 nM, MFe) and high (100nM, HFe) Fe supplementation concentrations through pairwise comparisons (i.e., HFe vs. LFe, MFe vs. LFe, and HFe vs. MFe). Transcript levels at each Fe supplement concentration are shown as log_2_ transformed read counts per million (CPM) and are centered and scaled according to the formula Z = (x-μ)/σ, where x is the mean of log_2_CPM in one treatment, μ is the mean of log_2_CPM values in all treatments, and σ is the standard deviation of the overall normal distribution curve.





**Figure S3.** Scatter plot of the differentially expressed genes (DEGs) between pairwise comparisons of Fe supplementation treatments. The genes are color-coded according to functions.


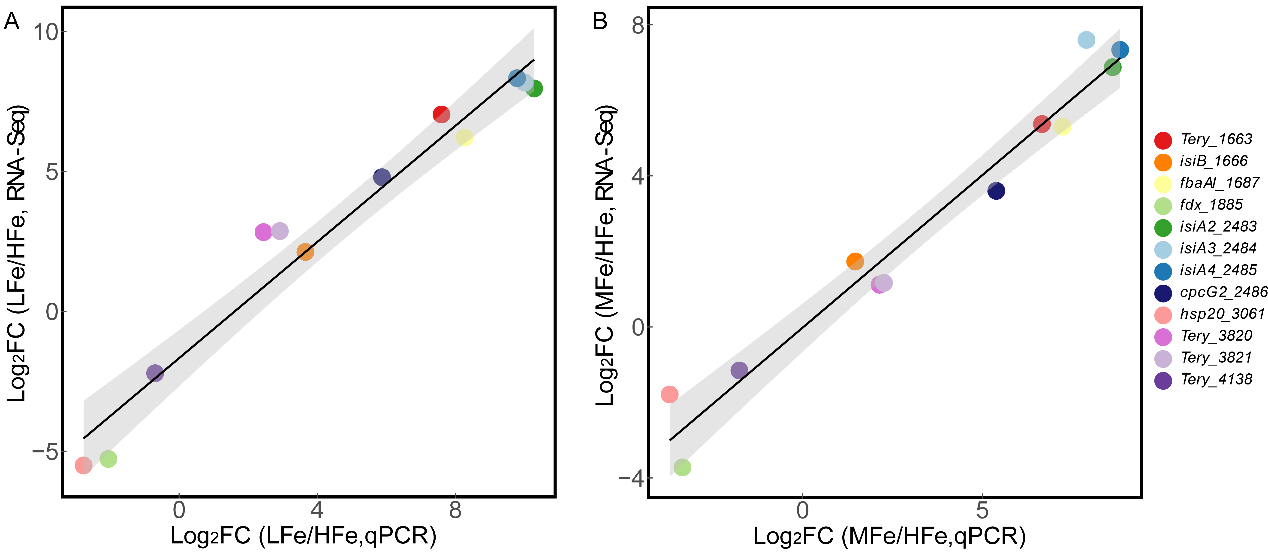


**Figure S4**: Verification of the RNA-Seq data with quantitative PCR (qPCR) assay. A total of 12 selected genes were analyzed with qPCR. The analysis showed significant correlation in relative transcript changes in the LFe/HFe (A) and MFe/HFe (B) comparisons between qPCR and the transcriptome sequencing results. Genes are labeled with gene symbol (if available) followed by locus tag of the open reading frame (e.g., *isiB_1666* denoting the *isiB* gene in the locus tag *Tery_1666* in the *Trichodesmium* genome).

**
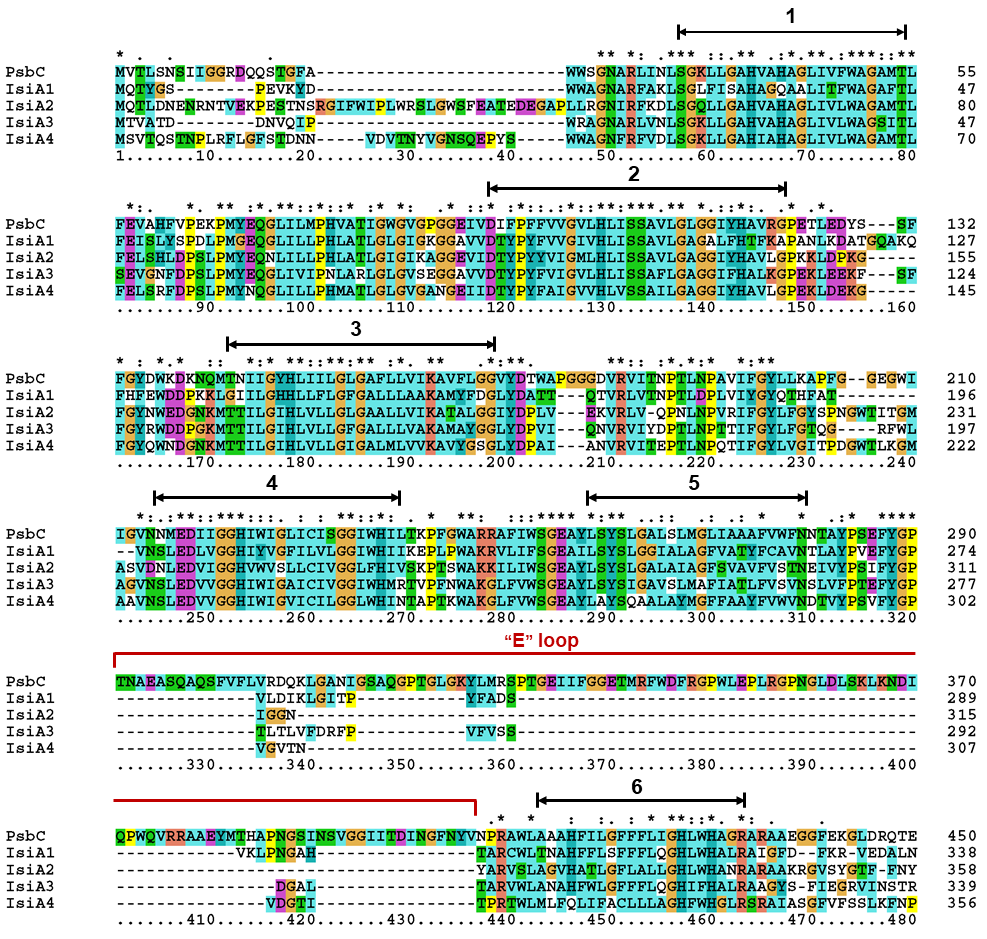
**

**Figure S5.** Alignment of IsiA1, IsiA2, IsiA3, IsiA4, and PsbC amino acid sequences in *T. erythraeum* IMS101. The six transmembrane regions are numbered and marked with double-arrowed lines. The huge loop “E” that is typical for PsbC (i.e., CP43) on the lumenal side between transmembranes 5 and 6 is indicated.


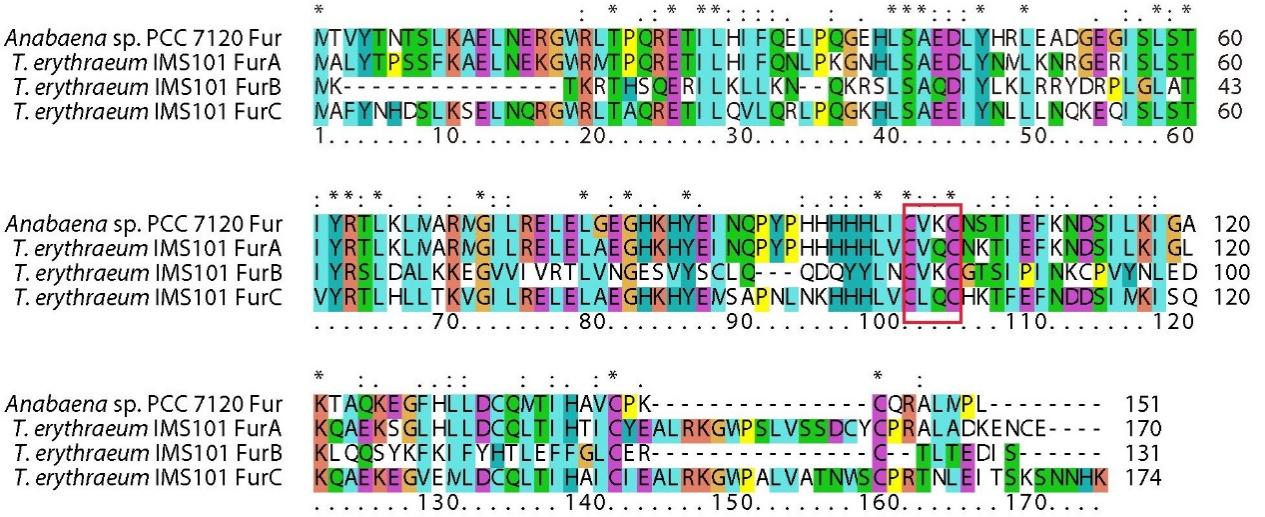


**Figure S6.** Alignment of the Fur family proteins in *T. erythraeum* IMS101 and *Anabaena* sp. PCC 7120. The ubiquitously conserved “CXXC” motif critical for the disulfide reductase activity for a Fur family member is boxed in red.

**
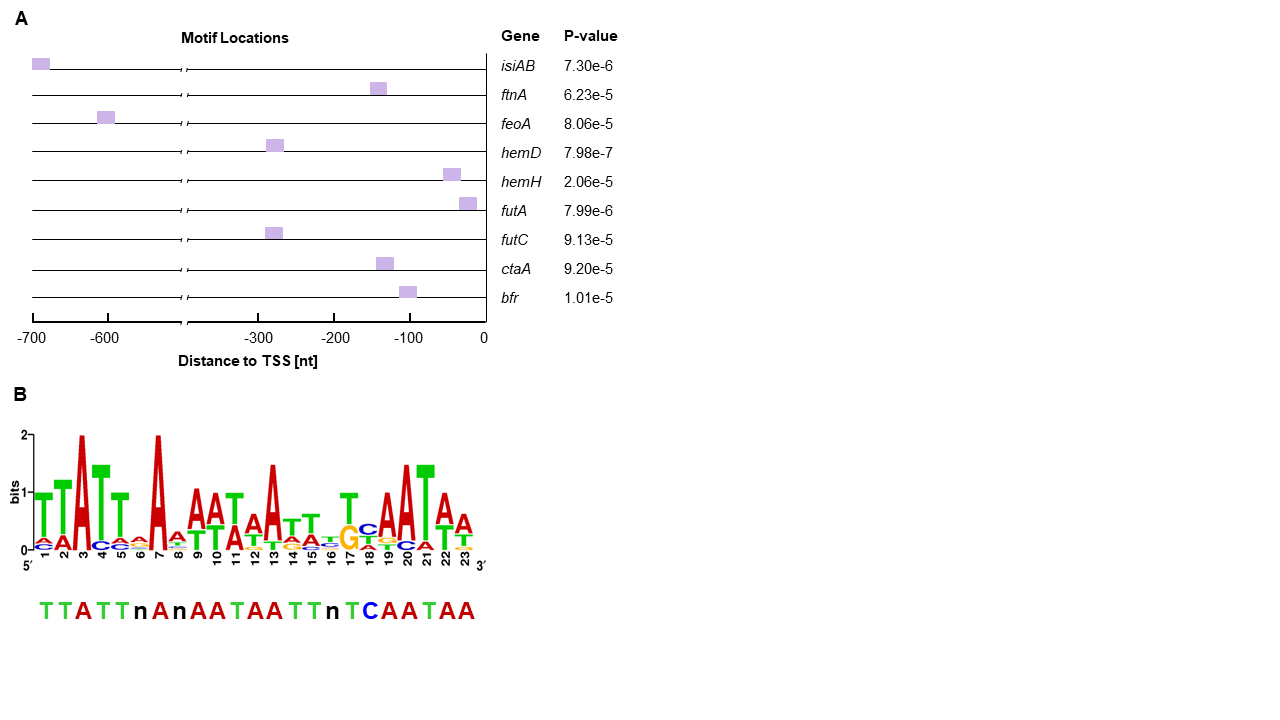
**

**Figure S7.** Putative Fur boxes in *T. erythraeum* IMS101. A) Locations of the Fur boxes (in purple) relative to the transcription start sites (TSS) of nine Fe-responsive genes in *T. erythraeum* IMS101. The Fur binding sites were predicted with the MEME program (Bailey et al., 2015) and an algorithm at <https://github.com/housw/GRPutils> (Hou et al., 2018). B) Sequence logos of the putative Fur boxes shown in A.

**References**

Bailey, T.L., Johnson, J., Grant, C.E. and Noble, W.S. 2015. The MEME Suite. Nucleic Acids Research 43(W1), W39−W49.

Hou, S., López-Pérez, M., Pfreundt, U., Belkin, N., Stüber, K., Huettel, B., Reinhardt, R., Berman-Frank, I., Rodriguez-Valera, F. and Hess, W.R. 2018. Benefit from decline: the primary transcriptome of Alteromonas macleodii str. Te101 during Trichodesmium demise. ISME J 12(4), 981−996.
